# The Frequency of Unhealthy Food Advertising on Mainland Chinese Television (TV) and Children and Adolescents’ Risk of Exposure to Them

**DOI:** 10.1371/journal.pone.0128746

**Published:** 2015-07-02

**Authors:** Zhenghua Zhou, Qinqin Diao, Nan Shao, Youke Liang, Li Lin, Yan Lei, Lingmei Zheng

**Affiliations:** Department of Nutrition and Food Hygiene, School of Preventive Medicine, North Sichuan Medical College, Nanchong, 637000, Sichuan, People’s Republic of China; Agricultural University of Athens, GREECE

## Abstract

**Objective:**

To conduct an analysis of the frequency of unhealthy food advertising on mainland Chinese television (TV) and children and adolescents’ risk of exposure to them.

**Methods:**

The frequencies of all types of advertisements (ads) on forty TV channels in mainland China, the exact ad broadcast times, and the name and brand of all snacks and western fast foods advertised were recorded from 0800 hours to 2400 hours on both a weekday and a weekend day in a week. The difference in the frequencies of the diverse types of ads over eight time intervals (each time interval was 2 hours) were compared, and the trends in ad frequencies during the time intervals were described.

**Results:**

The TV channels broadcast 155 (91-183) (expressed as median [*P*_25_-*P*_75_]) food ads, 87 (38-123) snack ads, 49 (11-85) beverage ads, and 58 (25-76) ads of snacks suitable for limited consumption (SSLCs) in a day. The proportion of snack ads among food ads (SPF%) was 55.5% (40.3%-71.0%), and the proportion of SSLC ads among snack ads (LPS%) was 67.4% (55.4%-79.3%). The ad frequencies for food, snacks, SSLCs, and beverages demonstrated significant differences among the eight time intervals (all *P*=0.000). TV channels broadcast the most frequent ads for food, snacks, SSLCs, and beverages during the time interval from 2000 hours to 2200 hours among the eight time intervals.

**Conclusions:**

Chinese children and adolescents may be at a high risk of exposure to unhealthy food advertising on TV. Reducing the exposure risk strongly requires multisectoral cooperation.

## Introduction

As a developing country, China has experienced rapid economic and social development along with cultural and lifestyle changes during the past decades. China’s nutrition transition has occurred at an accelerated pace compared with the changes experienced by other low- and middle-income countries [1–3]. Despite the prevalence of extensive undernutrition with respect to calcium, zinc, selenium, magnesium, thiamine, riboflavin [4], and vitamin A [5] among Chinese residents, the prevalence of overweight, general obesity and abdominal (or central) obesity among Chinese adults has increased markedly [6–7] since 1982, when overweight and obesity were observed to represent emerging conditions [8]. In the interim, the prevalence of overweight and obesity among Chinese children and adolescents has become increasingly common [9–13], which significantly threatens their health. In 2010, approximately 9.9% of Chinese school-aged children and adolescents (7–18 years old) were overweight and an additional 5.1% were obese, representing an estimated 30.43 million individuals [11].

A high prevalence of overweight and obesity increase cardiometabolic risks in Chinese children and adolescents [14–17], factors that are more likely to aggravate their future disease risk in adulthood. China will bear the substantial medical costs and the economic burden of overweight and obesity-related diseases if the epidemic continues to progress rapidly [18–20], suggesting an urgent need to control the obesity epidemic and prevent the development of chronic diseases.

The causes of overweight and obesity are multifactorial. In addition to genetic factors, environmental and behavioral factors are the important risk factors. In China, rapid economic growth and urbanization have promoted changes in food choices, a shift from physical to sedentary labor, and the creation of obesogenic environments, which affect physical activity levels, diet, behaviors and sociocultural norms. All these effects combine to create a state of positive energy balance that promotes obesity in children and adolescents [21–23].

A longer duration of television (TV) viewing is one of the risk behaviors for obesity [24–26]. TV viewing not only reduces energy expenditure by displacing physical activity but also increases the chances of exposure to food advertisements (ads). Several studies in western countries have demonstrated that TV viewing and exposure to TV food ads led children and adolescents to increase their food knowledge and to develop unhealthy food preferences, food habits, and eating behaviors [27–32] with subsequent increased consumption of the energy-dense low-nutrient foods frequently advertised on TV [33–35], which was likely to contribute to the prevalence of their overweight and obesity [36]. Additionally, the content analysis of TV food ads conducted in the United States [37], Australia [38–39], United Kingdom [40], Bulgaria [41], Turkey [42], nine other European countries [43], Singapore [44], South Korea [45], and Chinese Hong Kong [46–47] have consistently shown that children and adolescents are exposed to a large number of TV ads for energy-dense low-nutrient foods. However, there has not yet been a study analyzing the extent of TV food advertising in mainland China. Therefore, such an analysis is critical to understanding this component of the current obesogenic environment for Chinese children and adolescents.

On the other hand, Chinese Advertising Law and other relevant laws and regulations lack protection regulations for specific consumer groups, such as children and adolescents [48]. The Advertising Law of the People's Republic of China [49] and the Food Safety Law of the People's Republic of China [50] emphasize that advertisement content should be true and legal and must not contain any false, exaggerated content. The Advertising Law of the People's Republic of China [49] stipulates in article 8 that an advertisement shall not cause any damage to the physical and mental health of underage persons or handicapped persons. However, the stipulation is not operable in the judicial practice because it is relatively abstract. Nevertheless, the Food Safety Law of the People's Republic of China [50] and Chinese Food Advertising Regulatory System [51] do not have more specific stipulations for protecting children and adolescents, such as limiting the food ad frequency or restricting the broadcast time interval of food ads.

China Central Television & Sofres Media Research (CSM Media Research) is a joint venture between China Central Television Survey Center & Taylor Nelson Sofres (CVSC-TNS) Research and Kantar Media. Dedicated to TV and radio audience measurement research, CSM Media Research offers reliable and uninterrupted rating information for Hong Kong Special Administration Region and mainland China [52]. Data from CSM Media Research have shown that the sum of both the general rating market shares of China Central Television (CCTV) channels and the provincial satellite TV (PSTV) channels were 57.5%, 58.8%, and 61.0% during the first half of 2011, 2012, and 2013, respectively, and those of variety show programs were up to 80.6%, 78.6%, and 80.8%, respectively [53–54]. In 2012, PSTV strongly attracted the female, youth, and student populations, thereby yielding rating market shares of PSTV for audiences ages 4–24 years that were much higher than the average level for all audiences [55]. Notwithstanding, 9 CCTV channels and 10 PSTV channels were in the top 20 for channel coverage [55]. According to the Tabulation on the 2010 Population Census of the People’s Republic of China [56], nine CCTV channels and thirty-one PSTV channels covered 388,313,684 children and adolescents ages 4–24 years in 23 provinces, 4 autonomous regions, and 4 municipalities in mainland China, accounting for 29.15% of the total population (1,332,810,869 persons). These data indicated that a study on the CCTV and PSTV channels would be highly reliable and representative for the study on the frequency of unhealthy food advertising on mainland Chinese television (TV).

Snacks refer to all foods and drinks (not including water) consumed outside the context of the three main meals (breakfast, lunch, and dinner) and are referred to as morning, afternoon, and evening snacks, which constitute the ‘snack occasions’ [21, 57–58]. According to ‘Guidelines on snacks for Chinese children and adolescents (GSCCAs)’ [57], snacks were classified into ten groups: ‘sweet snacks’, ‘meat, seafood, and eggs’, ‘cereals and products’, ‘beans and products’, ‘vegetable and fruit’, ‘milk and products’, ‘nuts and seeds’, ‘tubers and products’, ‘beverages’, and ‘cold drinks’. The snacks were also classified by three ranks according to GSCCAs. Rank 1 included snacks that were suitable for regular consumption (SSRCs), which were mostly nutrient-rich, low-fat, low-salt, and low-sugar foods, such as yogurts, fruits, soybeans and sweet potatoes. Rank 2 was assigned to snacks that were suitable for moderate consumption (SSMCs), which were relatively nutrient-rich foods with an intermediate level of energy, fat, salt, and sugar, such as cookies and desserts. Rank 3 items were snacks that were suitable for limited consumption (SSLCs) that were high in energy, fat, salt, and sugar, such as instant noodles, candy, and deep-fried versions of puffed foods. SSLCs were also referred to as unhealthy, noncore foods [41, 46]. People who eat SSLCs frequently have a high risk of overweight, obesity, hypertension and other chronic diseases.

The aim of the present study was to conduct an analysis of the frequency of unhealthy food advertising on mainland Chinese television (TV) and children and adolescents’ risk of exposure to them. This study provides current data on the ad frequencies of unhealthy foods, including snacks and western fast foods, the trends in ad frequencies with time intervals, and the peak time of ad frequencies.

## Materials and Methods

### Ethics statement

This analysis of anonymised, aggregated data did not include individual human participants and, therefore, did not require ethical review from the Medical Ethics Committee of North Sichuan Medical College, according to the Biomedical Research Ethics Review Method Involving Human (Trial Edition) issued by the Ministry of Health of the People's Republic of China, now known as the National Health and Family Planning Commission of the People’s Republic of China (see: http://www.nhfpc.gov.cn/qjjys/s3581/200804/b9f1bfee4ab344ec892e68097296e2a8.shtml).

### Data collection

From July to August 2012, this study surveyed nine Chinese language CCTV channels and thirty-one PSTV channels. Each channel was surveyed from 0800 hours to 2400 hours on both a weekday and a weekend day within one week. The time monitored for the study period was 32 hours per TV channel and 1280 hours in total. While watching TV, the qualified investigators recorded the frequencies of all types of ads and the exact broadcast time, name, and brand of all the snacks and western fast foods advertised.

### Frequency of the diverse types of ads

The frequencies of all ad types (gross ads) and of ads for food, snacks, western fast foods, SSRCs, SSMCs, and SSLCs in each time interval (2 hours) from 0800 hours to 2400 hours and the frequencies of various food groups in snack ads on a weekday and a weekend day were counted. The frequencies of diverse types of ads on a day were calculated as the mean for those ads on a weekday and on a weekend day. Next, the ad frequencies were used to calculate the following ratios: food ads as a proportion of the gross ads (FPG%), snack ads as a proportion of the food ads (SPF%), snack ads as a proportion of the gross ads (SPG%), western fast food ads as a proportion of the food ads (WPF%), beverage ads as a proportion of the snack ads (BPS%), SSRC ads as a proportion of the snack ads (RPS%), SSMC ads as a proportion of the snack ads (MPS%), and SSLC ads as a proportion of the snack ads (LPS%).

### Description of peak time of ad frequency

The differences in the frequencies of the diverse types of ads among the time intervals were compared, and the trends of ad frequencies (median) with time interval and the peak time of ad frequencies (median) were described.

### Statistical analyses

Raw data were initially entered into the Tables Module of WPS Office 2012 Personal Version for Windows from Kingsoft Office Software Corporation (Beijing, People’s Republic of China) and processed as a classification of SFF ads with all parameters among groups. Statistical analyses of nonparametric tests were performed using SPSS version 17.0 statistical packages (Chicago, IL) for Windows. All parameters per day were expressed as the median (*P*_25_-*P*_75_) per TV channel. The difference among several related samples was analyzed by the Friedman test. A *P*-value <0.05 was considered statistically significant.

## Results

### Frequency and proportion of diverse types of ads

**Fig 1:**
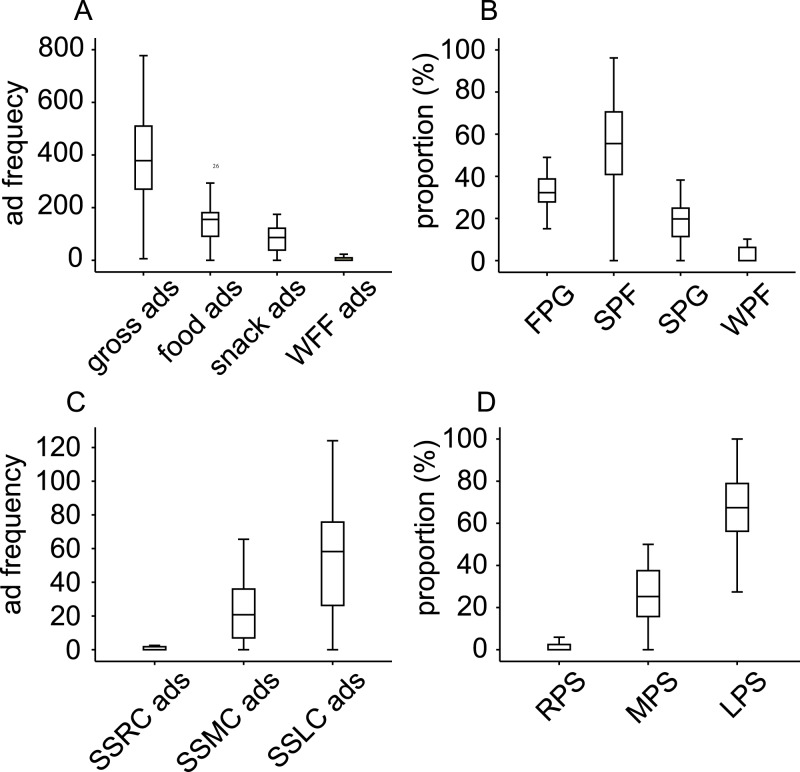
The ad frequency and proportion of diverse types of ads. WFF, western fast food; FPG, food ads as a proportion of the gross ads; SPF, snack ads as a proportion of the food ads; SPG, snack ads as a proportion of the gross ads; WPF, western fast food ads as a proportion of the food ads; BPS, beverage ads as a proportion of the snack ads; RPS, SSRC ads as a proportion of the snack ads; MPS, SSMC ads as a proportion of the snack ads; LPS, SSLC ads as a proportion of the snack ads; SSRCs, snacks suitable for regular consumption; SSMCs, snacks suitable for moderate consumption; SSLCs, snacks suitable for limited consumption. The sample was 40 TV channels.

Fig 1 shows the frequency and proportion of diverse types of ads. The TV channels broadcast 379 (268–511) gross ads, 155 (91–183) food ads, and 87 (38–123) snack ads, 0 (0–10) western fast food ads in a day. And the TV channels broadcast 0 (0–2) SSRC ads, 21 (7–36) SSMC ads, and 58 (25–76) SSLC ads in a day. The FPG% was 32.3% (27.5%-38.9%), SPF% was 55.5% (40.3%-71.0%), SPG% was 19.8% (11.2%-24.9%), WPF% was 0.0% (0.0%-6.3%), RPS% was 0.0% (0.0%-2.4%), MPS% was 25.2% (15.4%-37.5%), and LPS% was 67.4% (55.4%-79.3%).

### Frequency of various food groups in snack ads

The TV channels broadcast the following ten groups of snacks: ‘sweet snacks’, ‘meat, seafood, and eggs’, ‘cereals and products’, ‘beans and products’, ‘vegetables and fruit’, ‘milk and products’, ‘nuts and seeds’, ‘tubers and products’, ‘beverages’, and ‘cold drinks’. Table 1 shows that the frequencies of SSRC ads, SSMC ads, and SSLC ads were significantly different among the food groups in a week (all *P* = 0.000). Among all the food groups, beverage ads were broadcast by the TV channels at the highest frequency [49 (11–85)], with the frequency of SSLC beverage ads being 40 (7–62) and that of SSMC beverage ads being 9 (1–18). The PBS% was 67.1% (38.3%- 84.2%).

**Table 1:** Ad frequencies of various food groups of snacks on TV channels*.

Food groups	SSRC ads		SSMC ads		SSLC ads		Total		*P* value^‡^
	Median	*P*_25_-*P*_75_	Median	*P*_25_-*P*_75_	Median	*P*_25_-*P*_75_	Median	*P*_25_-*P*_75_	
Sweet snacks	0	0–0	0	0–0	1	0–11	1	0–16	0.000
Meat, seafood, and eggs	0	0–0	0	0–2	0	0–0	0	0–1	0.000
Cereals	0	0–0	0	0–11	1	0–4	3	0–14	0.000
Beans & products	0	0–0	0	0–0	0	0–0	0	0–0	0.368
Vegetable & fruit	0	0–0	0	0–0	0	0–0	0	0–0	0.607
Milk & products	0	0–1	0	0–1	0	0–1	1	0–5	0.437
Nuts & seeds	0	0–0	0	0–0	0	0–0	0	0–0	0.368
Tubers	0	0–0	0	0–0	0	0–3	0	0–4	0.000
Beverages	0	0–0	10	1–19	41	8–62	49	11–85	0.000
Cold drinks	0	0–0	0	0–0	0	0–0	0	0–0	0.050
Total	0	0–2	21	7–36	58	25–76	92	38–128	0.000
*P* value^†^		0.000		0.000		0.000		0.000	

SSRC, snack suitable for regular consumption; SSMC, snack suitable for moderate consumption; SSLC, snack suitable for limited consumption.

* The sample was 40 TV channels.

^†^ *P* value for difference among food categories by Friedman test for several related samples.

^‡^ *P* value for difference among SSRC, SSMC, and SSLC ads by Friedman test for several related samples.

### The peak time of ad frequency

**Fig 2:**
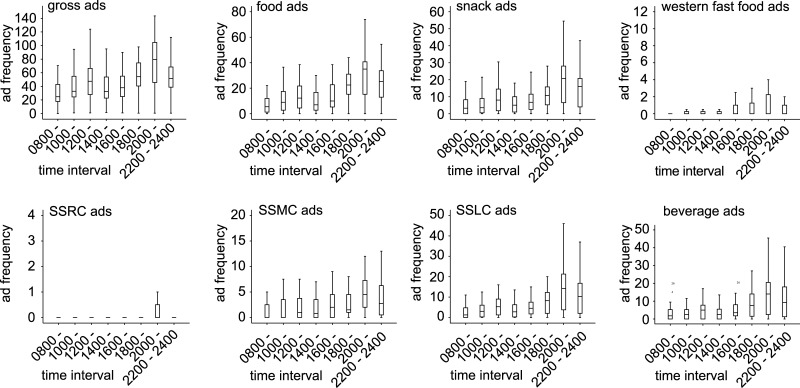
The trends in ad frequency during the time intervals. SSRCs, snacks suitable for regular consumption; SSMCs, snacks suitable for moderate consumption; SSLCs, snacks suitable for limited consumption. By Friedman test for several related samples, the difference was significant among time intervals in a day in gross ads (*P* = 0.000), food ads (*P* = 0.000), snack ads (*P* = 0.000), western fast food ads (*P* = 0.039), SSMC ads (*P* = 0.000), SSLC ads (*P* = 0.000), and beverage ads (*P* = 0.000). But the difference was not significant in SSRC ads (*P* = 0.275). The sample was 40 TV channels.

Fig 2 shows that the difference is significant among time intervals in a day for gross ads (*P* = 0.000), food ads (*P* = 0.000), snack ads (*P* = 0.000), western fast food ads (*P* = 0.039), SSMC ads (*P* = 0.000), SSLC ads (*P* = 0.000), and beverage ads (*P* = 0.000). And Fig 2 shows that the trends in ad frequency during the time intervals are described as double peaks at both the time intervals of 1200 hours to 1400 hours and 2000 hours to 2200 hours. The TV channels broadcast the highest frequencies of gross, food, snack, western fast food, SSMC, SSLC, and beverage ads during the time interval of 2000 hours to 2200 hours among the eight time intervals; the next highest frequencies were during the time intervals from 2200 hours to 2400 hours and from 1800 hours to 2000 hours.

## Discussion

In the present study, approximately one-third of ads were food ads, equivalent to the high levels observed in Bulgaria (33.4%) [41], Turkey (32.1%) [42], and Singapore (33%) [44], and higher than those in Australia in 2008 (15%) [39], United Kingdom (12.8%) [40], Brazil (11%) [46], and Greece (29%) [46]. In addition, approximately half of food ads were snack ads. The high frequency of food ads might facilitate children’s increased exposure to the food ads at the context that the high proportion of children received food ads information. A study showed that 68.69% of children 8–12 years old received food ad information, especially 43.86% of the children who were willing to take the initiative to receive food ad information [59]. The children’s high exposure to food ads might promote their high favorability regarding the advertised foods, deep memory of them and high desire to buy them. Under the influence of food advertising on TV, 78% of children would take action to consume snacks advertised, particularly the approximately 18% of children who frequently consume the snacks advertised [59], which might reflect the snacking status in China. One survey demonstrated that 14.4% of primary school students, 15.3% of junior high school students, and 21.3% of high school students in urban areas always or often snacked as they watched TV or used computers [60]. Since 2004, snacking has dramatically increased, which is one of the great shifts occurring in eating behaviors in the context of rapid economic growth and urbanization [21, 58]. In 2011, 56.7% of the population ages 2 years and older were reported to snack, including 67.1% of children 2–18 years of age. Moreover, the proportion of energy consumed from snacks was 9.5% among those who snacked and, notably, was 13.0% among children 2–18 years old. Higher proportions of energy consumed from snacks were observed with higher degrees of urbanization [21].

In the present study, SSLCs, also referred to as unhealthy, noncore foods [41, 46], were the most frequent subcategory represented among snack ads (median 67.4%). The proportion was higher than that in Australia in 2008 (49%) [39], United Kingdom (56%) [40], Singapore (57%) [44], and Hong Kong, China (49.2%) [47], and lower than that in Bulgaria (96.8%) [41], Turkey (84%) [42], and Germany (87%) [46]. The high frequency of SSLC ads might facilitate children’s increased exposure to the SSLC ads considering the high proportion of children who received food ad information [59], which may have effects on the unhealthy food preferences and eating behaviors of children and adolescents. Regular consumption of SSLCs may elevate the risk of obesity and related metabolic abnormalities among children [61].

Beverage ads were the most frequent among the food groups of snack ads, a finding that was validated against the data from CSM Media Research. Other than cosmetics and bathroom articles for daily use, beverages ranked first in the amount of TV advertising in China, and other foods ranked second in 2011 and 2012 [55, 62]. The high frequency of beverage ads may be associated with the rise in children’s consumption of beverages in China, similar to what has been observed in the United States [63]. The proportion of urban Chinese children who selected beverages as the most commonly consumed item increased from 59.5% in 1998 to 75.7% in 2008, and the average amount of beverage intake per day increased from 329.1 mL to 528.8 mL, respectively [64]. Most of the beverages for sale in China contain added sugars or sweeteners that supply a substantial amount of energy [65]. Furthermore, up to 16.1% of snacking energy intake was obtained from beverages among children ages 2–18 years in 2009 [58], which may be one of the risk factors for obesity, abdominal obesity and suicidal behaviors among Chinese children and adolescents [66–68].

Western fast food advertising might facilitate Chinese children and adolescents’ exposure to the SSLC ads, which might be one of the factors within obesogenic environments that influence eating behaviors and attitudes towards fast foods. A study by Ma et al. showed that the frequency of eating western fast foods among urban children and adolescents increased rapidly from 1998 to 2008 [69]. In 2009, 7.9% of snacking energy intake was from western fast foods among children ages 2–18 years [58]. Moreover, only 6.7% of children and adolescents in 2008 believed the western fast foods were unbalanced foods, and only 22.1% believed that western fast foods had low nutritional value [70]. Despite these findings, the relationship needs to be studied further.

The trends in ad frequencies over time intervals as displayed in Fig 2 were remarkably consistent with the general trends in TV ratings throughout the day in 2010, 2011, and 2012, particularly for the population ages 4–24 years [55]. The peak time for the ads for food, snacks, SSMCs, and SSLCs was from 1800 hours to 2200 hours, a period that is considered as prime time for Chinese TV, which increases the likelihood that Chinese children and adolescents will be exposed to the unhealthy food ads and will then increase consumption of the advertised unhealthy foods [33–34]. Interestingly, mainland Chinese children and adolescents prefer to eat snacks in the evening [58]. Thus, it is speculated that Chinese children and adolescents are eating more snacks while watching more snack ads during the peak evening TV-viewing period.

In summary, the high frequency of unhealthy food ads might increase the risk of exposure to these ads for Chinese children and adolescents, particularly during the evening prime time. The high risk of exposure to unhealthy food ads might promote their high favorability regarding the advertised foods, deep memory of them and high desire to buy them, representing potential risk factors for overweight and obesity for Chinese children and adolescents.

Multisectoral cooperation may be one strategy to reduce children’s exposure to unhealthy food advertising and to further decrease the effects of such exposure on overweight and obesity among children and adolescents. It is necessary and important for the various regulatory bodies to summarize the advertising regulations related to the experience of children and adolescents to improve food advertising legislation and to strengthen the food advertising regulatory system [48–51]. In an era of rapid agro-food industry growth in China, food companies have gained immense economic benefits. Notwithstanding, they should extend greater efforts to bear more environmental and social responsibility, including supplying healthier foods, engaging in self-regulation and voluntary initiatives to reduce children’s exposure to the characteristically misleading food advertising on TV and other media and increasing the effectiveness of nutrition labeling with accurate health claims [71–75]. Increased public health investment to implement certain nationwide campaigns, including social education, health education, and intervention programs [71], are also warranted.

## Conclusion

The present study presented the frequency of unhealthy food advertising on TV directed toward children and adolescents in mainland China. We demonstrated the high frequency of unhealthy food ads, especially beverages and SSLC ads, that were broadcast in a day, particularly during the evening prime time. The results indicated that Chinese children and adolescents may be at a high risk of exposure to unhealthy food ads on TV, which requires multisectoral cooperation to reduce this exposure risk.
